# First complete genome sequence and molecular characterization of *Canine morbillivirus* isolated in Central Brazil

**DOI:** 10.1038/s41598-021-92183-2

**Published:** 2021-06-22

**Authors:** Vivaldo Gomes da Costa, Marielena Vogel Saivish, Priscila Gomes de Oliveira, Abelardo Silva-Júnior, Marcos Lázaro Moreli, Ricardo Henrique Krüger

**Affiliations:** 1 Enzymology Laboratory, Department of Cell Biology, Universidade de Brasília, Distrito Federal, Brazil; 2 Department of Dermatological, Infectious and Parasitic Disease, Faculdade de Medicina de São José do Rio Preto, São Paulo, Brazil; 3 Veterinary Laboratory, Institute of Agricultural Sciences, Universidade Federal de Jataí, Goiás, Brazil; 4 Laboratory of Immunobiological and Animal Virology, Department of Veterinary, Universidade Federal de Viçosa, Minas Gerais, Brazil; 5 Virology Laboratory, Institute of Health Sciences, Universidade Federal de Jataí, Goiás, Brazil

**Keywords:** Microbiology, Diseases

## Abstract

The Brazilian regions are still highly endemic areas for *Canine morbillivirus* [canine distemper virus (CDV)]. However, little is known regarding the genetic variability of the strain circulating in several Brazilian regions. Here, we report the first full-length genome and molecular characterization of CDV isolated from domestic dogs in the Brazilian Center-West region. Sequence alignment and phylogenetic analyses based on deduced amino acid and nucleotide sequences showed that the isolated strain is characterized as the South America-I/Europe genotype. However, it segregates into a CDV subgenotype branch. Interestingly, both H and F proteins have a gain of a potential *N*-glycosylation sites compared to the Onderstepoort vaccine strain. Therefore, this study provides a reference to further understand the epidemic and molecular characteristics of the CDV in Brazil.

## Introduction

*Canine morbillivirus* [also known as canine distemper virus (CDV)] is a highly contagious and deadly pathogen of dogs and wildlife^1,2^. CDV, which belongs to the genus *Morbillivirus* in the *Paramyxoviridae* family, is enveloped with single-stranded, negative sense, and non-segmented RNA genetic material^3^. The principal mode by which dogs are infected is through airborne exposure to respiratory droplets carrying infectious virus or by direct contact of susceptible animals with the various fresh body secretions of a CDV-positive dog or wild animal. CDV infection can result in canine distemper (CD), with clinical signs including the onset of cutaneous rash, serious nasal and ocular discharge, conjunctivitis, and anorexia, followed by gastrointestinal and respiratory signs^1,4,5^. Although CD results in multisystemic clinical signs^6^, viral infection shows a high incidence of neurological complications, and neurological signs may be progressive, generating sequelae and an expectation for a poor prognosis^7,8^.

CDV is characterized by mutations, evolutionary changes with high genetic diversity, and eventual vaccine failure^9–12^. Regarding the complete viral genome, it contains 15,690 nucleotides (nt) and encodes six structural proteins, termed nucleocapsid (N), phospho (P), large (L), matrix (M), hemagglutinin (H), and fusion (F), and two accessory non-structural proteins (C and V). The glycoproteins H and F are inserted on the surface of the viral particle; they play a key role in adsorption and fusion, respectively, from the virion to the host cell. The M protein fills the space between the envelope and the ribonucleoprotein, thus contributing to viral morphology and the packaging and budding process in the host cell membrane. The N protein stands out because it encapsulates the genome and protects the genetic material. The L and P proteins are involved in viral RNA transcription and replication. Finally, the P, C, and V proteins play a role in RNA synthesis, aid in the transition from primary transcription to replication of the viral genome, and potentially influence messenger RNA (mRNA) synthesis, respectively^5,8,12^.

Currently, there are at least 17 major CDV genetic lineages, including America-1 to America-5, Europe Wildlife, Arctic, South Africa, South America-1/Europe, South America-1 to South America-3, Rockborn-like, and Asia-1 to Asia-4^5,10^. Due to its greater genetic variability, the H and F genes have been the main choices for determining CDV genetic lineages^13,14^. In addition, these classifications are related to the geographic origin where the lineages have been detected. While Brazil is considered endemic for CD, with high disease incidence rates^15^, there have been limited studies conducted in the country related to virus isolation and molecular characterization of the circulating wild-type strains. To date, no studies have examined the full-length genome to characterize the Brazilian CDV field. Therefore, to elucidate the genetic basis of the protein diversity of CDV, we conducted amino acid and nucleotide sequence analysis of a recent field isolate, with a focus on the H and F genes, which are the most suitable targets to investigate the CDV variability and evolution^5,13,14^.

## Methods

### Ethics statement

All animal procedures were approved by the Animal Care Committee of the Federal University of Goiás, Goiânia, Brazil (approval ID: 054/17). All experiments were performed in accordance with relevant guidelines and regulations. The owners of all animals signed informed consent forms approved by the ethics committee. The biological samples, from dogs showing clinical signs suggestive of CD, were collected from the Veterinary Hospital of the Federal University of Jataí and the Control Center of Zoonoses of the municipality of Jataí, located in the Center-West region of Brazil.

### Reverse transcription-polymerase chain reaction (RT-PCR)

The ocular/nasal specimens from dogs were collected with flocked swabs placed into 1 mL universal transport medium (UTM; Copan, Brescia, Italy). These samples in UTM were separated and used for the detection of viral RNA and cryopreserved for virus isolation. Initially, the RNA was extracted using a QIAamp Viral RNA commercial kit (QIAGEN, Hilden, Germany) according to the manufacturer’s specifications. Briefly, the method involved synthesis of a complementary DNA (cDNA) strand with a denaturation mix consisting of 1.0 µL (10 pmol/µL) random hexamers (Promega, Inc), 0.5 µL nuclease-free water (Thermo Scientific, Inc), and 8.5 µL total RNA; this mix was denatured at 70 °C for 5 min and immediately incubated on ice. The RT mix solution consisted of 4 µL 5X Reverse Transcriptase Buffer, 1.8 µL (50 mM) MgCl_2_, 1.7 µL (2.5 mM) dNTPs, 1 µL (20 units) RNase inhibitor, and 1.5 µL GoScript enzyme (Promega, Inc). The RT mix was added to the denaturation mix and reverse transcription was performed in a total volume of 20 µL in an Amplitherm PCR Thermal Cycler for 10 min at 25 °C followed by 90 min at 42 °C; the reaction was terminated by heating to 70 °C for 15 min. Following protocol adaptations reported by Castilho et al.^16^ and Frisk et al.^17^, reverse transcription, PCR, and nested PCR were performed for the purpose of partial detection of the CDV N gene. After the addition of the possible amplicons in the 1.2% agarose gel stained with SYBR Safe DNA gel stain (Invitrogen, Carlsbad, CA, USA), the amplification product was visualized under ultraviolet light. The molecular identity of the expected PCR product (287 base pairs [bp]) was confirmed by DNA sequencing.

### Cell culture and virus isolation

VerodogSLAM (VDS) cells were obtained from the Department of Veterinary, Laboratory of Immunobiological and Animal Virology, at the Federal University of Viçosa, Brazil. VDS cells were maintained in Dulbecco’s Modified Eagle Medium (DMEM, D7777, Sigma-Aldrich, St. Louis, USA) supplemented with 10% fetal bovine serum (FBS) and 1% penicillin/streptomycin/amphotericin B (Vitrocell, Campinas, Brazil). Cells were cultured at 37 °C in a humidified incubator containing 5% CO_2_. In addition, zeocin (R25001, Gibco) antibiotic was added (1% final concentration) for stable maintenance of canine signaling lymphocytic activation molecule (SLAM) tag expression, which is one of the cellular receptors for CDV.

An aliquot of cryopreserved PCR-positive swab samples for CDV was thawed and filtered through a 0.22-μm filter and used as an inoculum for virus isolation. Thus, CDV isolation was attempted using VDS cells. Consequently, confluent VDS cells in 6-well plates were washed twice with phosphate-buffered saline (1X) and inoculated with 400 µL sample and 200 µL DMEM. After 1–2 h incubation with gentle shaking, more DMEM supplemented with 2% FBS and 1% antibiotic was added to each well. Inoculated cells were incubated at 37 °C with 5% CO_2_. The cell culture plate was observed each day under an inverted microscope to determine whether a cytopathic effect (CPE) developed. If there was a CPE, the supernatant was collected and confirmed to be positive for CDV by RT-PCR. All positive cell cultures were subjected to molecular typing with whole genome sequencing. However, if no CPE was observed 7 days post-inoculation, the supernatants were inoculated on new VDS cells for a second passage. Finally, if the CPE tests and RT-PCR results were negative after three passages, the virus isolation result was considered negative.

### Primer design, RT-PCR and whole genome sequencing

CDV-specific primers (Supplementary Table S1) were designed based on the reference sequences for the target species, which were selected from an analysis of complete sequences accessed through the Virus Pathogen Database and Analysis Resource (ViPR)^18^ and nucleotide sequences available in GenBank^19^.

The primers were designed using Geneious Primer (2020.2)^20^ to cover the complete genome sequence of all CDV strains. The primers were designed to have a melting temperature (Tm) between 52 and 68 °C and not to form hairpin loops or primer dimers. In addition, the NCBI BLAST tool was used to confirm the specificity of the primers for CDV. Supplementary Table S1 shows the primers created and used for full‐length cDNA amplification and sequencing.

Viral RNA, extracted from cell lysate plus supernatant, was quantified by Qubit 4 Fluorometer. Briefly, RT reaction was carried out in a volume of 20 µl. Eight point five microliters of viral RNA plus 1.5 µl of Random primer (0.75 µg) were heated for 5 min at 70 °C and chilled on ice (5 min). Ten microliters of the previous reaction was mixed with 4 µl of 5 × buffer, 1.7 µl mix dNTP (2.5 mM), 1.8 µl of MgCl_2_ (20 mM), 1 µl of RNase inhibitor (20 units/µl) and 1.5 µl of RT (160 units/µl, GoScript, Promega, Inc.) were added. The mix was incubated for 5 min at 25 °C. Again, the mix was incubated for 90 min at 42 °C. The RT enzyme inactivation step was for 15 min at 70 °C.

**Figure 1:**
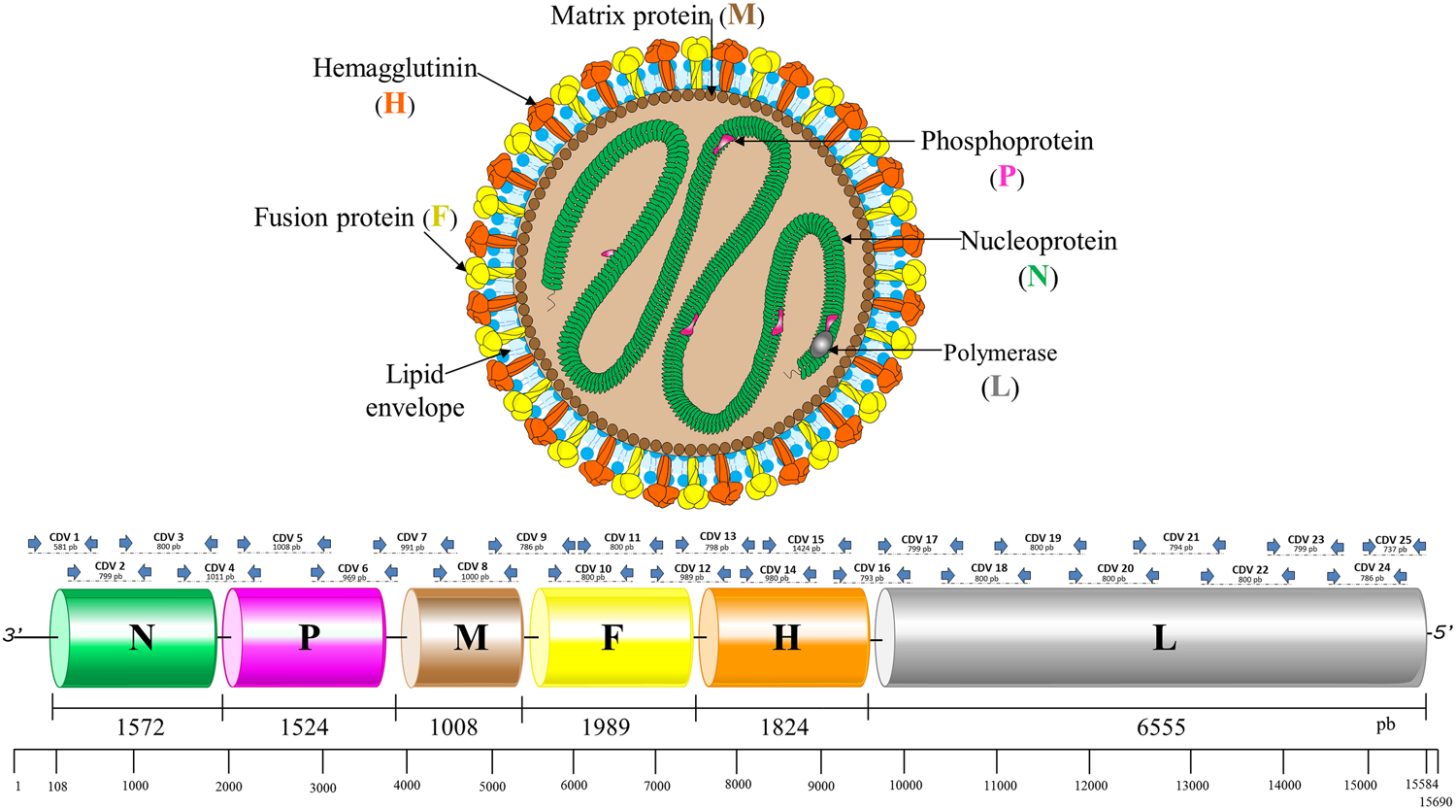
Schematic organization of the canine distemper virus (CDV) genome and primer design.

PCR was conducted in a reaction volume of 50 µl containing 5 µl 10 × PCR buffer (Invitrogen), 2 µl MgSO_4_ (50 mM), 4 µl mix dNTPs (2.5 mM), 1 µl forward primer (10 µM), 1 µl reverse primer (10 µM), 0.4 µl Taq DNA polymerase High Fidelity (2 U) (Invitrogen), 28,6 µl nuclease-free water and 8 µl of template DNA. The initial denaturation at 94 °C for 2 min was followed by 50 cycles at 94 °C for 15 s, 58 °C for 30 s and 68 °C for 75 s. Taq DNA polymerase High Fidelity was used to amplify the genome in 25 overlapping fragments (Fig. 1). The PCR products were then purified with the QIAquick PCR purification kit according to the manufacturer’s protocol. Purified amplicons were sequenced bidirectionally using an ABI3.500 genetic analyzer (Applied Biosystems).

### Phylogenetic analysis and molecular characterization of CDV

Phylogenetic analysis was performed using the nucleotide/amino acid sequence, as well as the sequences of 24 reference strains for which full genome sequences were available in GenBank and ViPR. Sequences were edited and aligned using the Multiple Sequence Comparison by Log-Expectation (MUSCLE) program in the Geneious software package. A phylogenetic tree was constructed, based on the open reading frame (ORF) sequences of CDV, using the neighbor-joining method in the Geneious software package. Bootstrap analysis was carried out on 10,000 replicate data sets.

Potential *N*-glycosylation sites of H and F proteins were predicted using the NetNGlyc 1.0 Server (www.cbs.dtu.dk/services/NetNGlyc/)^21^.

Selection pressure on the F/H proteins was evaluated using four methods: *S*ingle *L*ikelihood *A*ncestor *C*ounting (SLAC), *F*ixed *E*ffects *L*ikelihood (FEL), *M*ixed *E*ffects *M*odel of *E*volution (MEME), and *F*ast *U*nconstrained *B*ayesian *A*pp*R*oximation, for inferring selection (FUBAR) on the Datamonkey web server^22^. A *p* value less than 0.05 for MEME and FEL and a posterior probability higher than 0.9 for FUBAR were considered suggestive of positive selection. The *G*enetic *A*lgorithm for *R*ecombination *D*etection (GARD) analysis in Datamonkey was performed to detect the recombination breakpoints in the H gene alignment of the wild-type CDV isolated in this study.

## Results

### Detection of the N gene in clinical specimens

**Figure 2:**
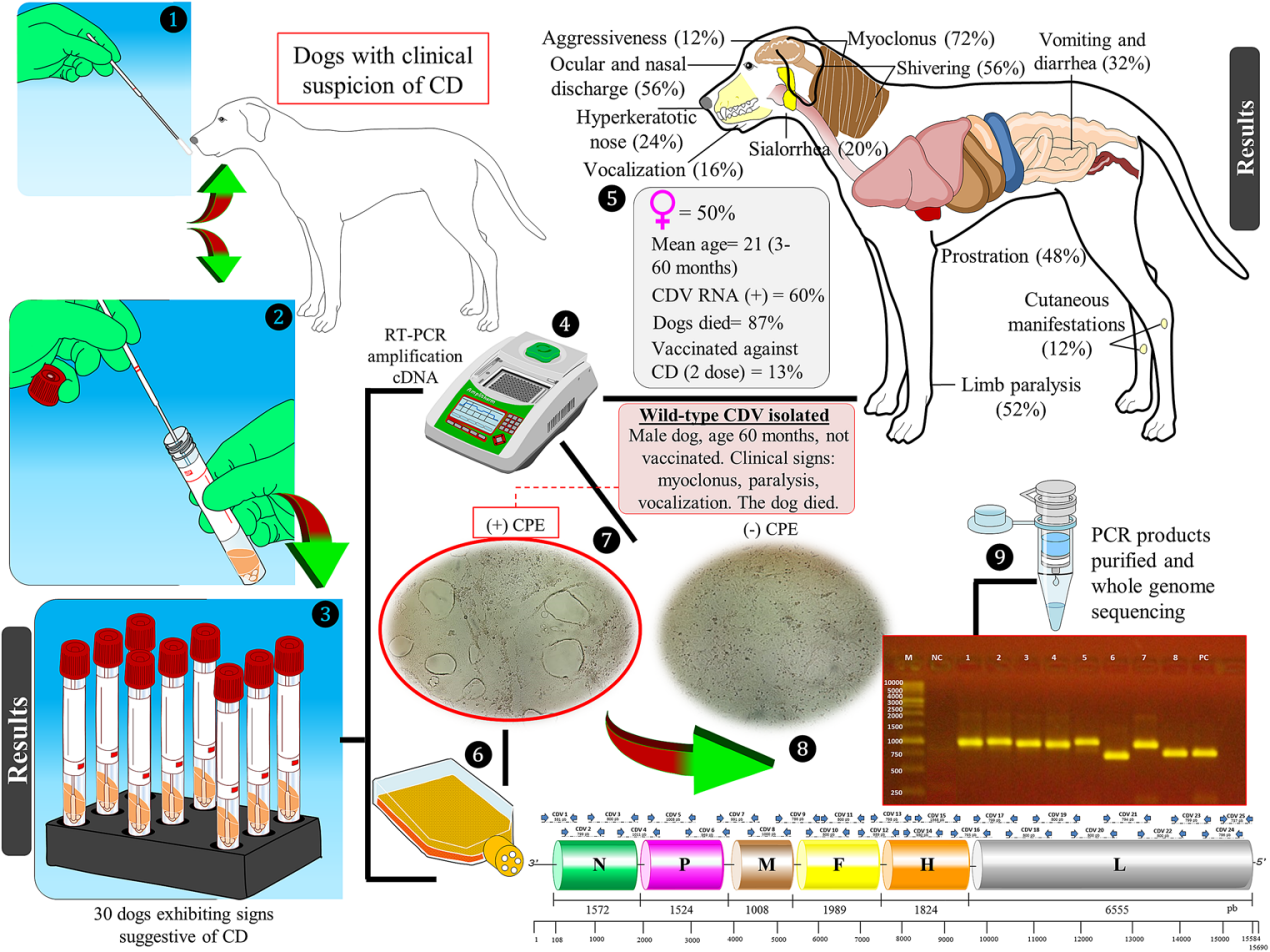
This schematic diagram reflects the experimental workflow. (1–3) In 2019, samples were collected from 30 domestic dogs showing clinical signs suggestive of canine distemper (CD). (4 and 5) Canine distemper virus (CDV) RNA (N gene) was detected from nasal samples in 60% of dogs. Also shown is the frequency of clinical signs for dogs that were CDV RNA positive. (6 and 7) Virus isolation was confirmed based on cell culture [one sample caused a cytopathic effect (CPE)] as well as reverse transcription–polymerase chain reaction (RT-PCR). (8 and 9) Subsequently, degenerate primers were generated to sequence the isolated CDV genome. 1.2% Agarose gel electrophoresis image [lanes contain PCR products from cDNA extracted from dog’s nasal swab (lane M, pb DNA standard marker), (lane NC, negative control), (lane 1–4, CDV 4–7 primers), (lane 5–8, CDV 12/25/14/17 primers), (lane PC, positive control)].

Biological samples from a total of 30 dogs with clinical suspicion of CD were collected in 2019. Preliminary identification of CDV was done using nested RT-PCR targeting the conserved region of the N gene. A total of 18 samples were positive and showed a specific band at 287 bp in an agarose gel. VDS cells were then inoculated with the samples. Gross lesions such as detachment of cells and the syncytial effect were observed. Again, the nested RT-PCR was employed on the harvested VDS cells to confirm the isolation of a virus, designated JA88/2020. In the third passage of JA88, a confluent CPE (~ 80%) was observed at 48 h post-infection. The concentration of viral RNA obtained from cell lysates plus supernatant was 74.8 ng/µl. Figure 2 presents the results with the synthesized information.

### Complete genome phylogenetic analysis

**Figure 3:**
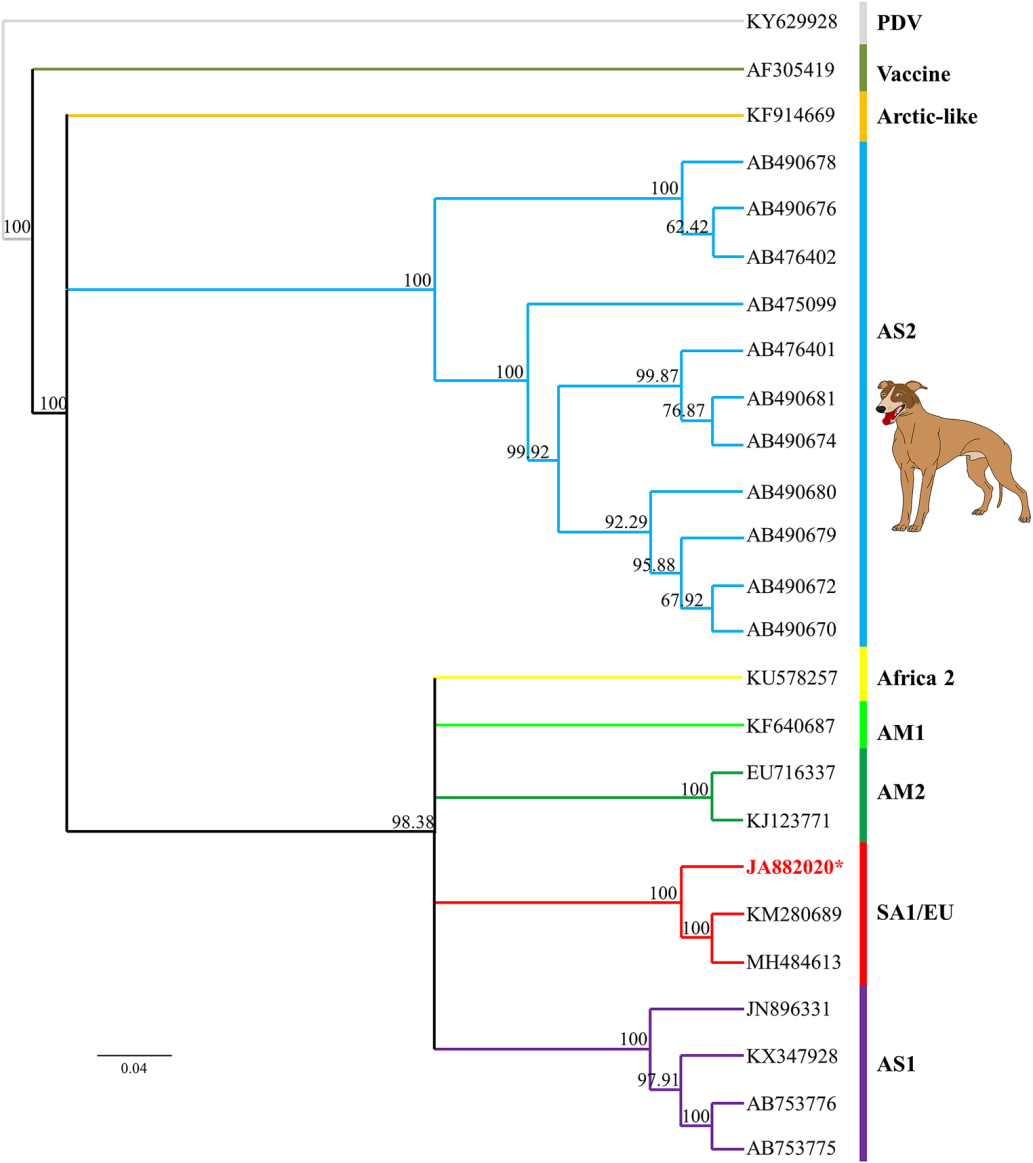
A phylogenetic tree based on amino acid sequences between the detected CDV and reference strains. Bootstrap values (> 50%) are shown at each node of the tree using 10,000 replicates. The scale bar below the tree represents a genetic distance of 0.04 amino acid substitutions per site. The CDV isolate identified in this study is indicated in red and with an asterisk. Sequences are labelled using GenBank accession numbers. SA1/EU: South America 1/Europe; AM1: America 1; AM2: America 2; AS1: Asia 1; AS2: Asia 2; PDV: Phocid distemper virus (was included as an outer group).

The JA88/2020 genome was fully sequenced (Fig. 2), and the complete nucleotide sequence has been deposited in GenBank under accession number MW460905. The whole genome sequencing comprises 15,624 nt with 100% consensus obtained by Sanger sequencing. The RNA contains six ORFs at 42–1613 nt (N gene); 1735–3258 nt (P gene); 3366–4373 nt (M gene); 4869–6857 nt (F gene); 7013–8836 nt (H gene); and 8964–15,518 nt (L gene). The 5′ untranslated region (UTR) and 3′-UTR are 106 and 41 nt long in the viral RNA, respectively. In addition, to identify the type of the CDV detected, the strain sequence was compared with those of 24 reference strains. Based on the standard criteria for classification of CDV lineages (nucleotide and amino acid sequence identity), JA88/2020 is part of the South America 1/Europe (SA1/EU) lineage: Its complete coding sequence displays 97.4% nucleotide and 98.5% amino acid similarity with the prototype strain Uy251 (Fig. 3). Interestingly, the genome-wide phylogenetic tree revealed that the strain isolated in this study segregates into a cluster of previously reported CDV lineages.

**Figure 4:**
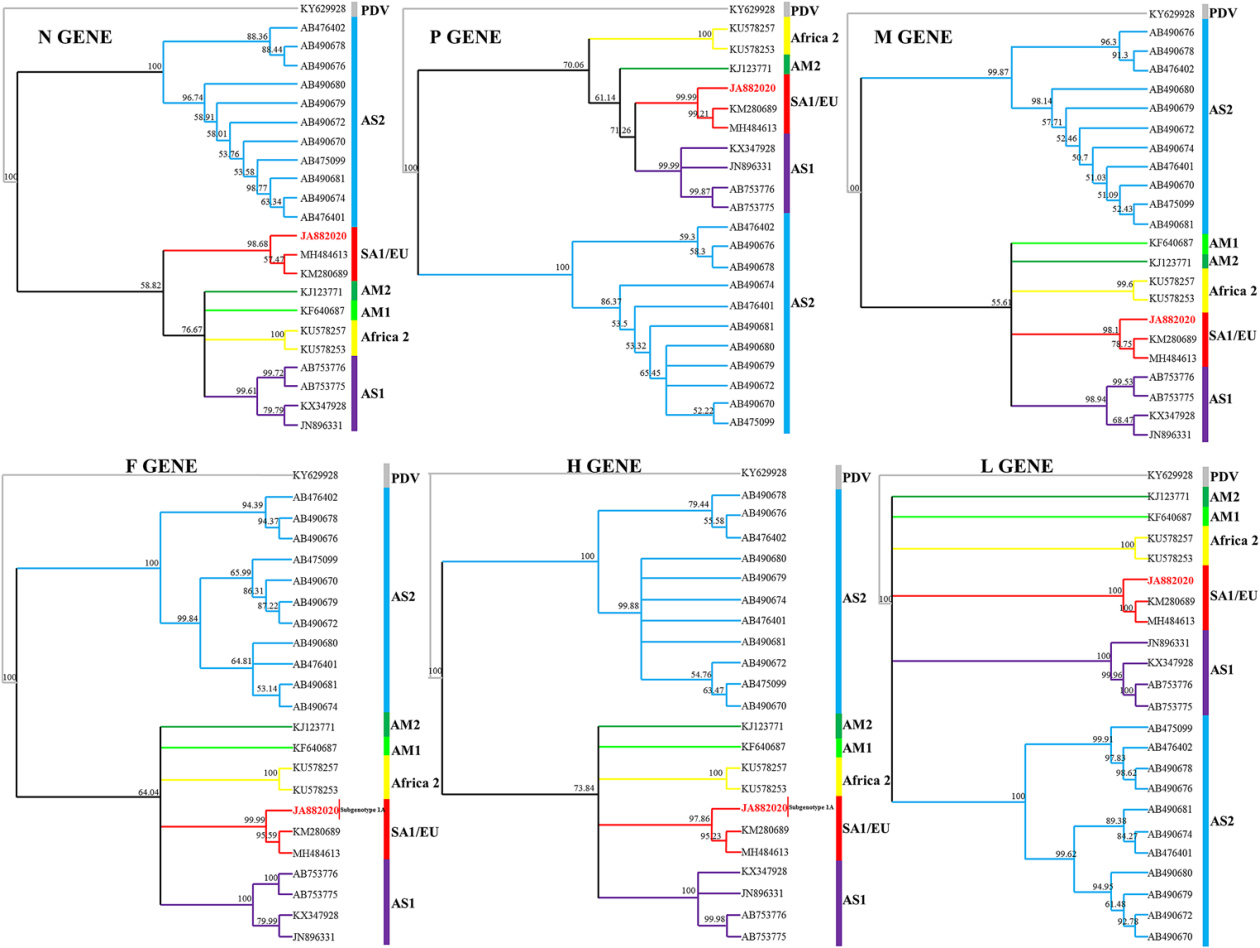
Phylogenetic relationships between CDV strains based on N, P, M, F, H, and L gene sequences. Bootstrap values are shown at each node of the tree. The phylogenetic tree was constructed by the neighbor-joining method using 10,000 bootstrap replicates. The CDV isolate identified in this study is indicated in bold.

Phylogenetic trees of each CDV gene (N, P, M, F, H, and L) were also made. The topology of trees has branch positioning similar to that of the genome (Fig. 4). When comparing each individual gene between JA882020 and SA1/EU, the nucleotide/amino acid identities are as follows: 98%/99.2% (N gene), 98%/98.6% (P gene), 97.12%/99.1% (M gene), 96.7%/97.3% (F gene), 97%/96% (H gene), and 97.2%/99.2% (L gene).

### CDV subgenotype and amino acid analysis of the H and F proteins

Following the criteria of at least 95% amino acid identity to define a genotype and 98% to define a CDV subgenotype, for H amino acid identity, we identified a subgenotype in the SA1/EU lineage (subgenotype 1A; 3.95% amino acid variation). Regarding the F amino acid identity, for the Fsp fragment gene sequences, we arbitrarily extrapolated the classification and also found a subgenotype within the SA1/EU lineage (subgenotype 1A; 4.4% amino acid variation).

**Figure 5:**
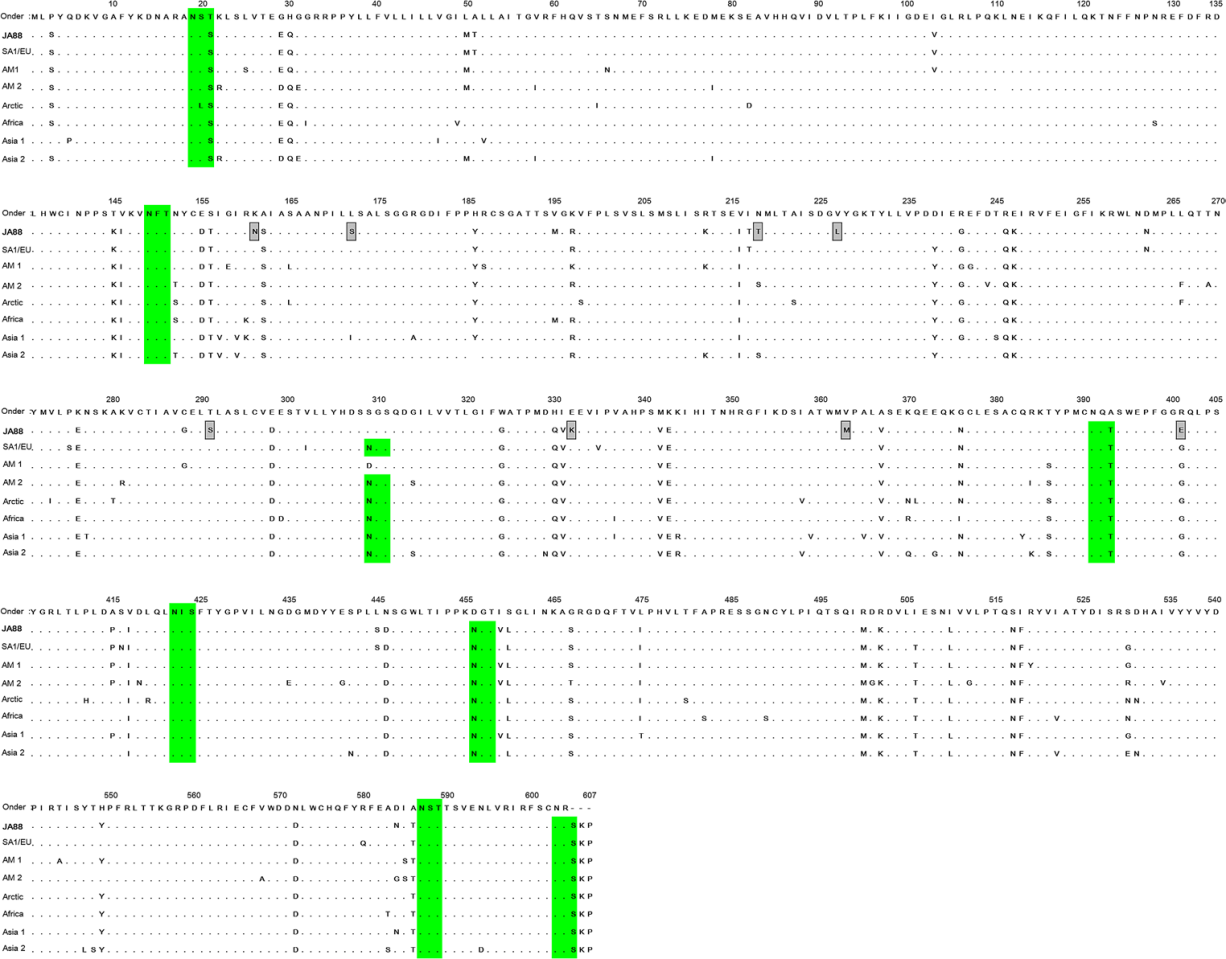
Amino acid sequence alignment of the CDV H protein from the Onderstepoort vaccine strain (AF305419); a wild-type strain from Brazil (JA88); and seven wild-type strains from Uruguay (SA1/EU: KM280689), the United State of America (AM1: EU716337; AM2: AY542312), Italy (Arctic: KF914669), South Africa (KY971528), China (AS1: JN896331), and Japan (AS2: AB490670). The gray box indicate amino acid residues unique to JA88 strain. The green region represents motifs associated with potential N-glycosylation sites (N-X-S/T). Dots (·) indicate identity.

**Figure 6:**
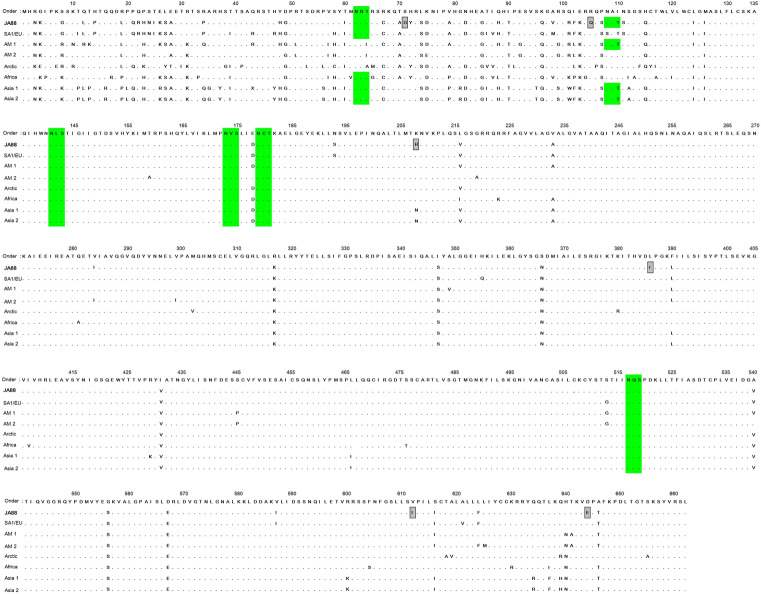
Amino acid sequence alignment of the CDV F protein from the Onderstepoort vaccine strain; a wild-type strain from Brazil (JA88); and seven wild-type strains (SA1/EU: KM280689; AM1: EU716337; AM2: AY542312; Arctic: KF914669; South Africa: KY971528; AS1: JN896331; AS2: AB490670). The gray box indicate amino acid residues unique to JA88 strain. The green region represents motifs associated with potential N-glycosylation sites (N-X-S/T). Dots (·) indicate identity.

The deduced full-length H and F amino acid sequences were aligned with the Onderstepoort vaccine strain and with wild-type strains from other parts of the world (Figs. 5 and 6). For the H protein, there are 59 amino acid variations. In the H amino acid sequences, eight substitutions at positions 161, 172, 218, 227, 291, 332, 363, and 401 are specific to the new JA88/2020 isolate. For the F protein, there are 62 amino acid variations. In the F amino acid sequences, six substitutions at positions 71, 105, 208, 386, 612, and 644 are specific to the new JA88/2020 isolate.

### H and F protein *N*-glycosylation sites analysis

Seven *N*-glycosylation sites are predicted at amino acid residues in the H protein: 19, 149, 391, 422, 456, 587, and 603. Our analysis also showed that these are conserved sites for *N*-glycosylation in the H protein for the SA1/EU, America 1/2, Africa, Asia-1/2, and Arctic lineages. Surprisingly, a potential *N*-glycosylation site at position 309 is lost in JA88/2020 compared with the other lineage from SA1/EU. Regarding the F protein, there are six *N*-glycosylation sites at amino acid residues 62, 108, 141, 173, 179, and 517, which are common sites for other lineages. In addition, an *N*-glycosylation site at amino acid residue 108 is lost in the reference lineage from SA1/EU (Uy251).

### Selection pressure and recombination within CDV

Selection pressure analysis of the F protein from all CDV strains revealed 18 positively selected codon sites. Of the 18 positive sites, three were detected by several methods (Supplementary Table S2). Codons 71, 208, 612, and 644, potential positively selected codon sites, were detected by FEL. Codons 21, 87, and 101 were detected by FUBAR and MEME. Codons 61 and 302, potential negatively selected codon sites, were detected by FEL. One hundred codon sites predicted under negative selection were detected by FUBAR (Supplementary Table S2). Finally, recombination events for codons 549–662 were found for CDV by analysis with GARD from the Datamonkey package.

Selection pressure analysis of the H protein revealed 14 positively selected codon sites. Of the 14 positive sites, three were detected by several methods (Supplementary Table S2). Codons 172, 218, 227, 291, 309, 401, and 530, potential positively selected codon sites, were detected by FEL. Codon 530 was detected by FEL, FUBAR, and MEME. Seventy-five codon sites predicted to be under negative selection were detected by FUBAR (Supplementary Table S3).

## Discussion

In this work, we amplified the complete coding and intergenic regions of the JA882020 SA1/EU strain obtained from a dog’s nasal swab. The sample came from a 5-year-old male that showed clinical manifestations of myoclonus, paralysis, and vocalization; the animal ultimately died. Importantly, degenerate primer sets were generated to sequence this isolate. Furthermore, we contribute to the identification of sequence variability, and this information is also valuable for selecting appropriate primers and excluding false-negative PCR results.

The putative natural recombination events of the CDV F gene have been reported by our group, and the results in this study correlate with other previous findings^23^. Recombination events have also been reported in the H gene^10,24–26^. Consequently, the introduction of genetic mutations and recombination result in significant genetic variability of RNA viruses and may lead to the emergence of new viral lineages. Therefore, it is necessary to monitor these events to understand the genetic evolution of CDV.

One of the main benefits of monitoring mutations in infectious agents is to associate whether possible non-synonymous mutations are contributing to the prevalence of a more contagious and/or pathogenic strain. It is worth mentioning the molecular epidemiological surveillance of two glycoproteins on the CDV viral surface: H and F. To evaluate the possibility that key residues are involved in virulent CDV, Zipperle et al.^27^ identified the key residues in the H protein (Y525, D526, and R529) that are involved in controlling SLAM-binding activity. SLAM and nectin-4 are CDV host cell receptors, which are expressed on activated T and B lymphocytes; epithelial, glial, and dendritic cells; and macrophages^5^. Consequently, based on the amino acid mutations of the viral isolate in this study, these key residues (H protein) have been conserved. However, it is important to search for new key residues in other proteins such as F to understand factors involved in virulent CDV.

Better molecular characterization of the CDV epidemic in Brazil is needed because CDV infection in dogs is high and deadly. Few studies have analyzed molecular epidemiology and carried out molecular analysis of full-length genes from Brazilian CDV lineages^28,29^. Therefore, some researchers have performed complete sequence analysis of the full-length F and H genes^14,30^. These reports have demonstrated the predominance of one genotype in Brazil: SA1/EU. However, two co-circulating lineages have already been detected, including the South America-II lineage. Moreover, similar results to our study have been described regarding CDV subgenotypes found in biological samples from dogs in Brazil and elsewhere^30,31^. CDV genotypes possibly differ due to geographic distribution rather than by host species. In this context, for a country as large as Brazil, concomitant circulation of different CDV genotypes is possible. Given this possibility, extensive molecular epidemiological surveys are required to determine the circulating (sub)genotypes^32^.

The unique molecular signatures of the F and H genes were identified through visual inspection from amino acid positions (F: S71G, R105Q, K208R, L386I, V612I, D644E, H: K161N, L172S, N218T, V227L, T291S, E332K, V363M, R401E). The analysis of polymorphisms featuring has been carried out by Fischer^28^; with the observation of unique amino acid signatures of the distemper epidemic in local dog populations from Rio Grande do Sul state, Brazil. Similarly, another study showed unique amino acid patterns for viral isolates from Argentina^11^. Here, we note that JA88 can be classified into a subgenotype (1A) based on the genetic diversity of the F and H genes. Consequently, the unique molecular signatures contributed to this finding. Further research will be important to ascertain whether these findings are stable in local dog populations, and/or whether they are involved in the gain of some characteristic of the virus, such as an increase or reduced virulence in these viral strains.

The *N*-glycosylation sites in the F and H proteins are essential for their correct folding, transport, and cell surface expression. Hence, it is necessary to ask what is the importance of monitoring *N*-glycosylation sites in viral proteins? Among the possible answers, the following stand out: in previous studies, researchers have hypothesized that reduced *N*-glycosylation contributes to attenuate CDV pathogenesis, and that an increase in *N*-glycosylation may eventually result in vaccine failure^33,34^. Here, we observed an extra putative *N*-glycosylation site in the F protein compared with the Uruguay sequence (Uy251). Strikingly, four additional *N*-glycosylation sites in the F and H proteins were found compared with the vaccine strain, thus showing its importance for modulating virulence.

In conclusion, this study is unique because it is the first that has isolated and identified the full CDV genome from Brazil. In sum, the isolated strain is characterized as the SA1/EU genotype, but it is segregated into a CDV subgenotype branch. Sequence analysis of more CDV field isolates from different Brazilian geographic regions is needed to investigate differences between (sub)genotypes. In addition, immunological investigation might be required to determine and monitor the biological relevance of circulating CDV (sub)genotypes and their importance for future drug and new vaccine development.

## Supplementary Information

Supplementary Information.
