# Supplementary material for: First complete genome sequence and molecular characterization of Canine morbillivirus isolated in Central Brazil
Source: Sci Rep. 2021 Jun 22;11:13039. doi: 10.1038/s41598-021-92183-2 (PMC8219677; doi:10.1038/s41598-021-92183-2)
Supplement: Supplementary file 1 — Supplementary Information. [file 41598_2021_92183_MOESM1_ESM.pdf]

Supplementary data

First complete genome sequence and molecular characterization of *Canine morbillivirus* isolated in Central Brazil

Vivaldo Gomes da Costa[1,*], Marielena Vogel Saivish[2,*], Priscila Gomes de Oliveira[3¶], Abelardo Silva-Júnior[4¶], Marcos Lázaro Moreli[5,*], Ricardo Henrique Krüger[1¶]

[1]Enzymology Laboratory, Department of Cell Biology, Universidade de Brasília, Distrito Federal, Brazil,

[2]Department of dermatological, infectious and parasitic disease, Faculdade de Medicina de São José do Rio Preto, São Paulo, Brazil,

[3]Veterinary Laboratory, Institute of Agricultural Sciences, Universidade Federal de Jataí, Goiás, Brazil,

[4]Laboratory of Immunobiological and Animal Virology, Department of Veterinary, Universidade Federal of Viçosa, Minas Gerais, Brazil.

[5]Virology Laboratory, Institute of Health Sciences, Universidade Federal de Jataí, Goiás, Brazil,

[¶]These authors contributed equally to this work.

*Corresponding authors: Universidade de Brasília, Distrito Federal, Brazil. Phone/Fax: + 55 64 99611-9241, Email address: vivbiom@gmail.com (VGdC); Faculdade de Medicina de São José do Rio Preto. E-mail: Email address: marielenasaivish@gmail.com (MVS); Universidade Federal de Jataí, Goiás, Brazil. Phone/Fax: + 55 64 99257-5959, Email address: marcos_moreli@ufg.br (MLM).

Table S1. Primers used for RT-PCR amplification of the CDV genome

Primer	Nucleotide sequence (5'-3')	Position	bp[1]	Gene(s)
CDV 1F	MCYTAGDGRACAAGGTCAGG	72-91	581	N
CDV 1R	ACTGCTTTAGCGAGCARKAT	652-633		
CDV 2F	TCAACTCTGTTTGYGGTCTT	430-449	799	N
CDV 2R	CCRRCRGATCTTCTRACCAT	1228-1209		
CDV 3F	TGAAACRGCRCCGTAYATGG	1028-1047	800	N/P
CDV 3R	GACATGRTAGGCCTGYTCYTC	1827-1807		
CDV 4F	GCTGGTCCCAAGCAATCTCA	1344-1363	1011	N/P
CDV 4R	ACATCAGCTGCTCTGTCTGG	2354-2335		
CDV 5F	GCGGTGAAGAGGTTAAGGGA	2156-2175	1008	P
CDV 5R	ATCCGATTGCCGAGCTAGAC	3163-3144		
CDV 6F	ACATTAACCCAGAGCTCCGC	2975-2994	969	P/M
CDV 6R	TCGTCTGATAGTCGAGTGATGC	3943-3922		
CDV 7F	CGAACTGCAGGTGTCAAGGA	3737-3756	991	M
CDV 7R	AGGCGAAGTTCAAAACCCCA	4727-4708		
CDV 8F	CAGCGATGATCAGGGTCTTT	4415-4434	1000	F
CDV 8R	GATCTTATAATGGACACTGTCAGTC	5414-5390		
CDV 9F	GAGWCCRRDACCTCCCRRGS	5019-5038	786	F
CDV 9R	RACYCCCTGAACRGCAATGA	5804-5785		
CDV 10F	CGTTTTGCAGGAGTGGTRC	5604-5622	800	F
CDV 10R	TTGTTGCCCATMGTYCCAGAY	6403-6383		
CDV 11F	YGTCTCAGARTCAGCMATTTG	6278-6298	800	F/H
CDV 11R	GYTGGACTACYTGAGCCCTA	7077-7058		
CDV 12F	CTGGTCACACGTCTTACCCG	6939-6958	989	F/H
CDV 12R	GGCTTTGGAATTCTCCGGGA	7927-7908		
CDV 13F	TYGGCAGCAAAHCCYATCM	7571-7589	798	H
CDV 13R	MACYGGACCRTATGTAAAYGAK	8368-8347		
CDV 14F	TGACCGCTATCTCAGACGGA	7746-7765	980	H
CDV 14R	AGAAATCGTCCGGATTGGGT	8725-8706		
CDV 15F	YTGTCTRGAGTCDGCTTGTC	8206-8225	1424	H/L
CDV 15R	TGATTCACCTYTYACAAAGACAGGR	9629-9605		
CDV 16F	YATGCRAAGCTCACARTGGTTC	9494-9515	793	L
CDV 16R	AGGAGATGCATGGACAGGRA	10286-10267		
CDV 17F	MGCAGCAGARAATGTAMGGA	10124-10143	799	L
CDV 17R	ACTRCTYGGRGARGAGACGG	10922-10903		
CDV 18F	AAGAAAGACTCMCATCGBGGY	10827-10847	800	L
CDV 18R	GCTCGGGTCTCRTCCACTA	11626-11608		
CDV 19F	AGCCTTRAGACAGAGRTTRCATGA	11444-11467	800	L
CDV 19R	TYGCACCHGTGATYGTGTT	12243-12225		
CDV 20F	CCYATGCTGAAAGGAYTRTTCCAT	12111-12134	800	L
CDV 20R	CTCTTGTCYCTKAGTCGRTGAGC	12910-12888		
CDV 21F	CWACYGTGTACTCRTGGGCCT	12574-12774	794	L
CDV 21R	TCCAGARGGYCGGTGATARTG	13547-13527		
CDV 22F	CYGCDTTAATKGGYGAYGAYG	13408-13428	800	L
CDV 22R	ARCYAGRTCATCTTTHGGTGG	14207-14187		
CDV 23F	RAGRAGAGGBTCWATYAAGCAGA	14051-14073	799	L
CDV 23R	ACTTCKMAGAAAATGTGGGAGTG	14849-14827		
CDV 24F	TGGAAGARCTGTCTGCYATA	14710-14729	786	L
CDV 24R	TKGGGATTGTYGTCAGGATKA	15495-15475		
CDV 25F	CAACAGATTTTGCGAGTCGGT	14961-14981	737	L
CDV 25R	CAGACAAAGCTGGGTATGATAACT	15697-15674		

[1]Amplified fragment. The accession numbers (GenBank) of CDV genome reference sequences used in order the design the primers were as follows: AF305419.1; MH484613.1; EU716337.1; JN896331.1; KF914669.1; AB476402.1; AB490676.1; AB475099.1; AB476401.1; AB490681.1; AB490674.1; AB490680.1; AB490679.1; AB490678.1; AB490670.1; AB490672.1; KU578257.1; KF640687.1; KM280689.1; KJ123771.1; KX347928.1; AB753776.1; AB753775.1.

Table S2. Codon sites predicted under positive selection with p-value threshold of 0,1

Gene	FEL	MEME	FUBAR
F	71, 208, 612, 644	21, 53, 87, 98, 101, 105, 112, 311, 354, 546, 654	21, 87, 101
H	172, 218, 227, 291, 309, 401, 530	218, 349, 386, 471, 500, 530	530

Table S3. Codon sites predicted under negative selection

Gene	FEL	FUBAR
F	61, 302	9,18, 66, 78, 83, 93, 103, 109, 119, 124,129, 133, 138,140, 147, 149, 150, 162, 168, 172, 176, 180, 196,197, 209,212, 218, 222, 229, 230, 239, 251, 258, 267, 292, 296, 298, 300, 306, 308, 315, 331, 332, 335, 350, 351, 356, 361, 362, 365, 370, 378, 380, 382, 386, 389, 394, 402, 403, 410, 412, 421, 435, 440, 441, 442, 451, 464, 465, 477, 483, 485, 486, 490, 495, 499, 506, 521, 523, 529, 530, 531, 533, 536, 538, 545, 549, 554, 569, 613, 623, 625, 633642, 645, 647, 650, 651, 655, 657
H		6, 9, 40, 62, 75, 80, 81,106, 112, 129, 132, 134, 141, 144, 147, 154, 162, 177, 180, 226, 245, 253, 264, 278, 285, 290, 300, 320, 322, 331, 339, 343, 347, 351, 352, 353, 380, 393, 398, 400, 406, 410, 414, 423, 424, 427, 429, 433, 452, 453, 454, 456, 464, 474, 476, 481, 492, 494, 518, 520, 528, 533, 536, 554, 556, 558, 566, 570, 571, 572, 576, 578, 581, 589, 598, 603

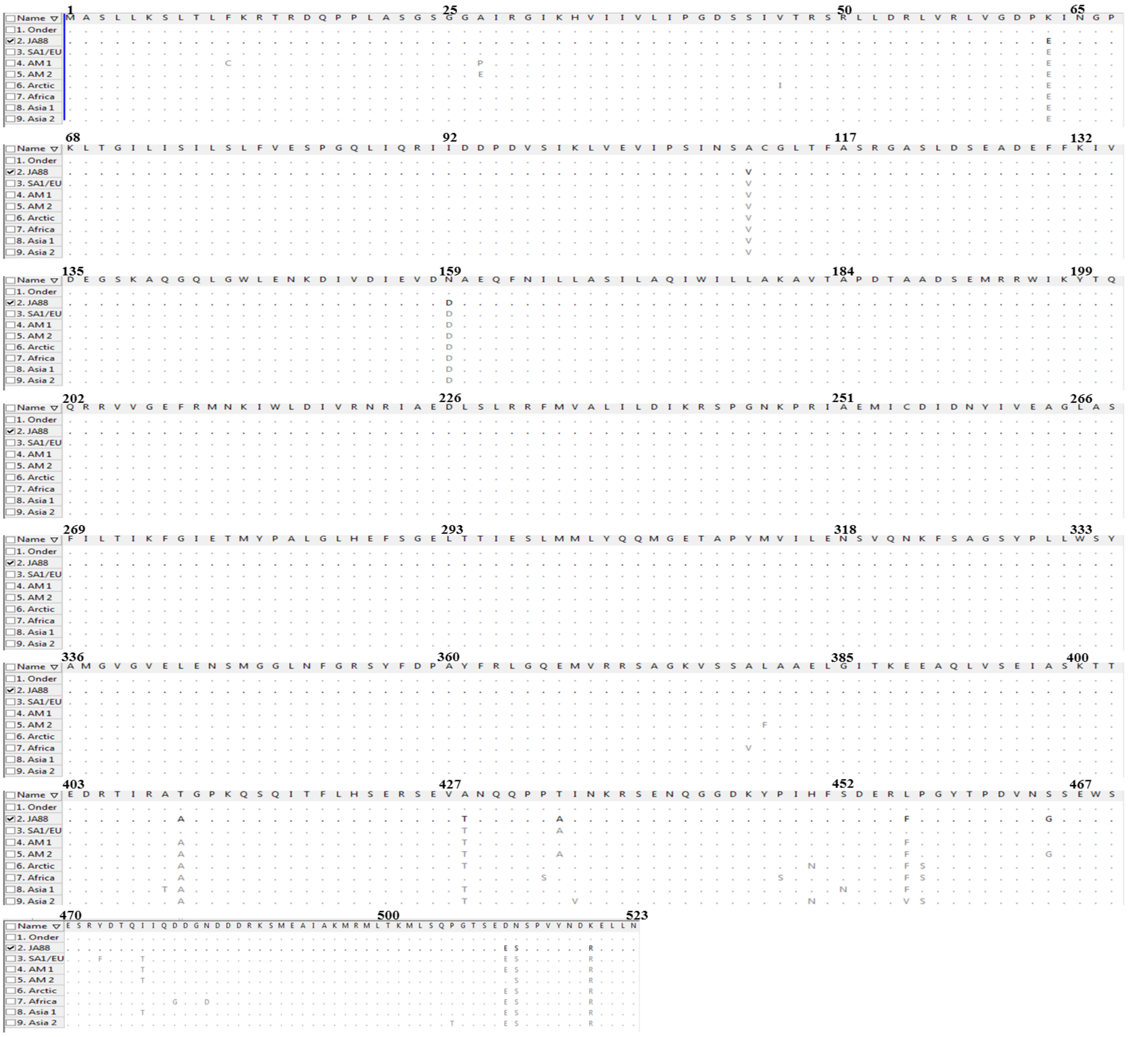

Fig. S1. Multiple amino acid sequences alignment of the Nucleoprotein. The sequences were compared by Clustal Multiple Sequence Alignment Program software. Dots (•) indicate identity.

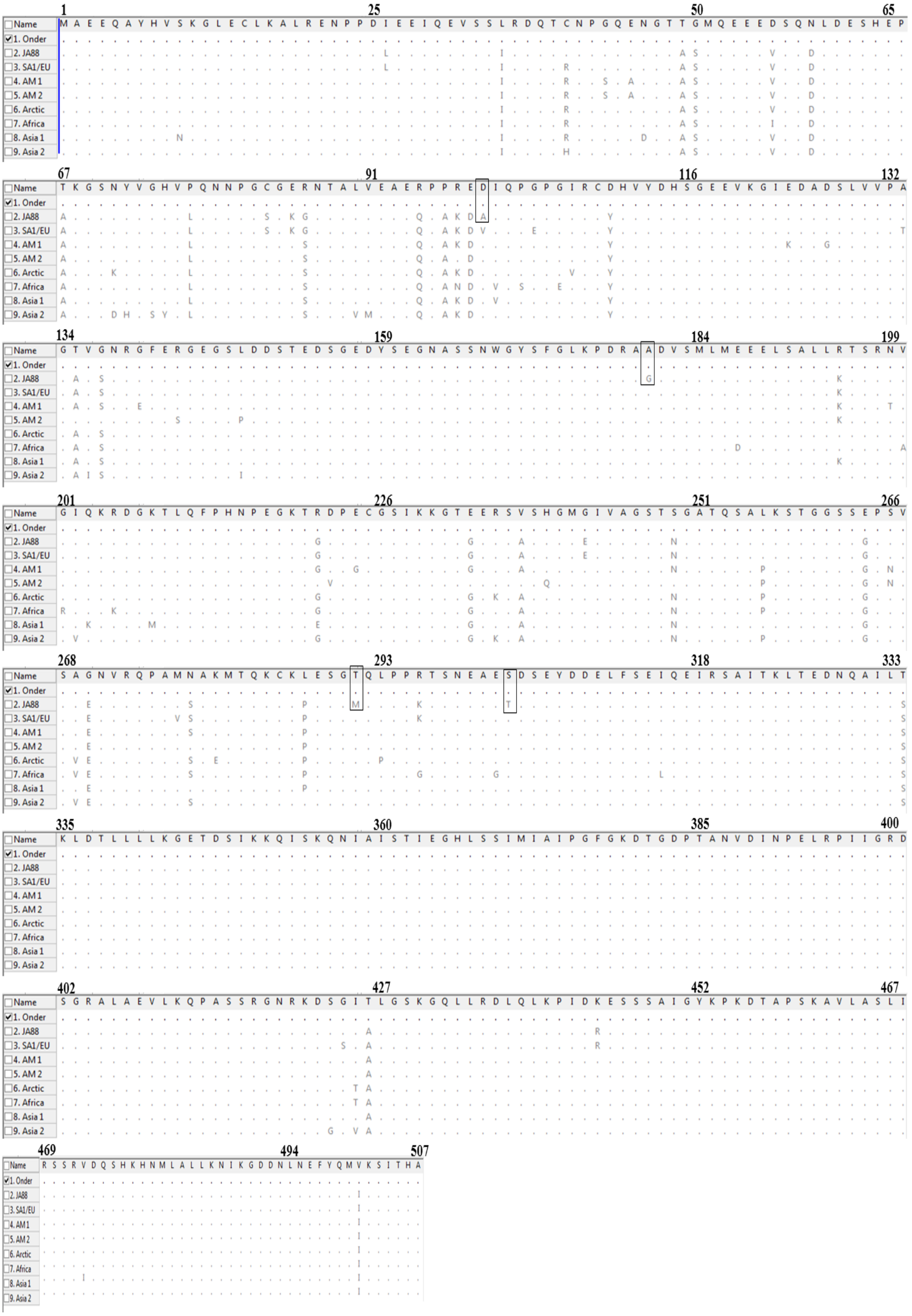

Fig. S2. Multiple amino acid sequence alignment of the Phosphoprotein. The sequences were compared by Clustal Multiple Sequence Alignment Program software. The boxes indicate amino acid residues unique to JA88 strain. Dots (•) indicate identity.

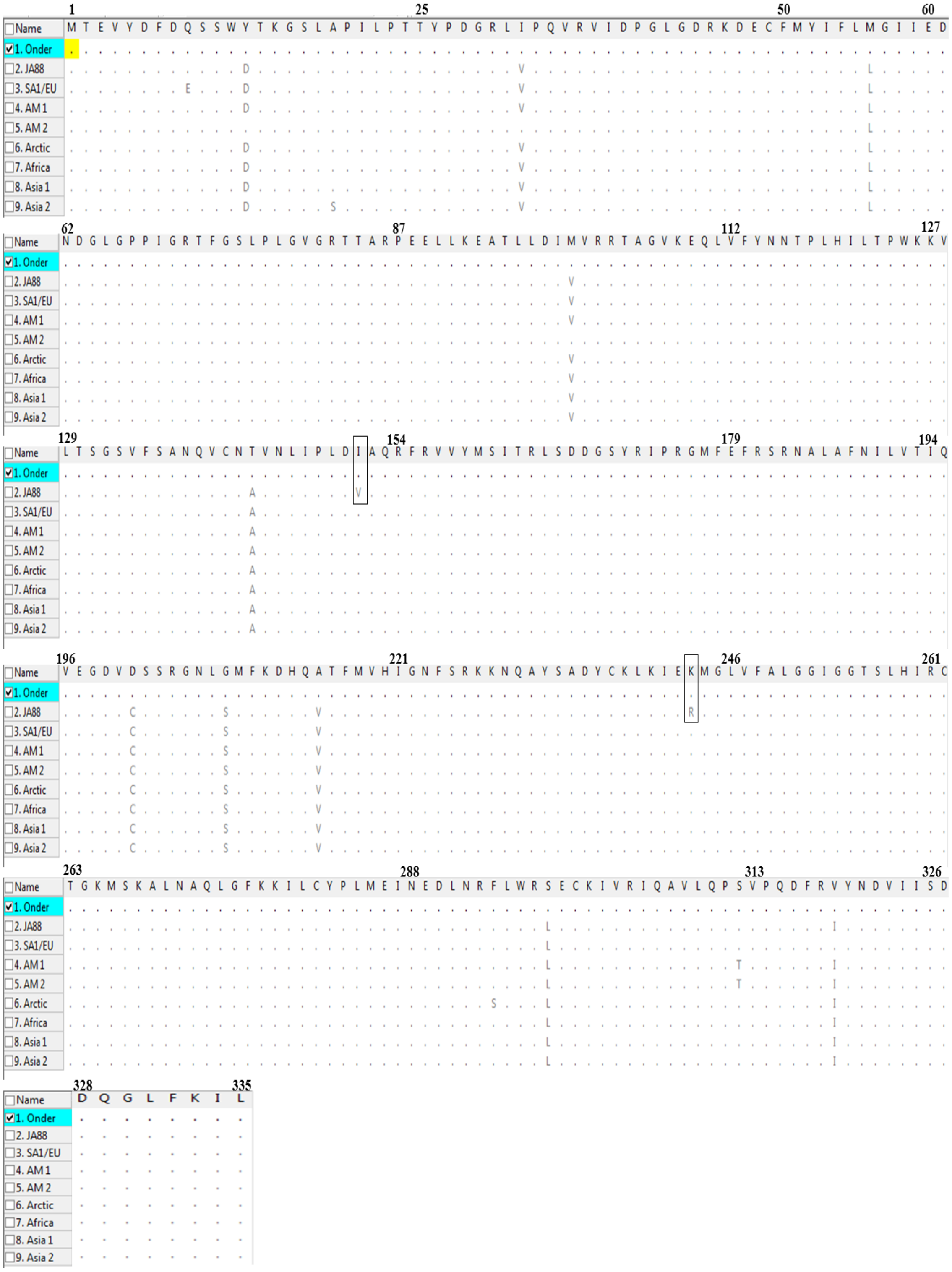

Fig. S3. Multiple amino acid sequence alignment of the Matrix protein. The sequences were compared by Clustal Multiple Sequence Alignment Program software. The boxes indicate amino acid residues unique to JA88 strain. Dots (•) indicate identity.

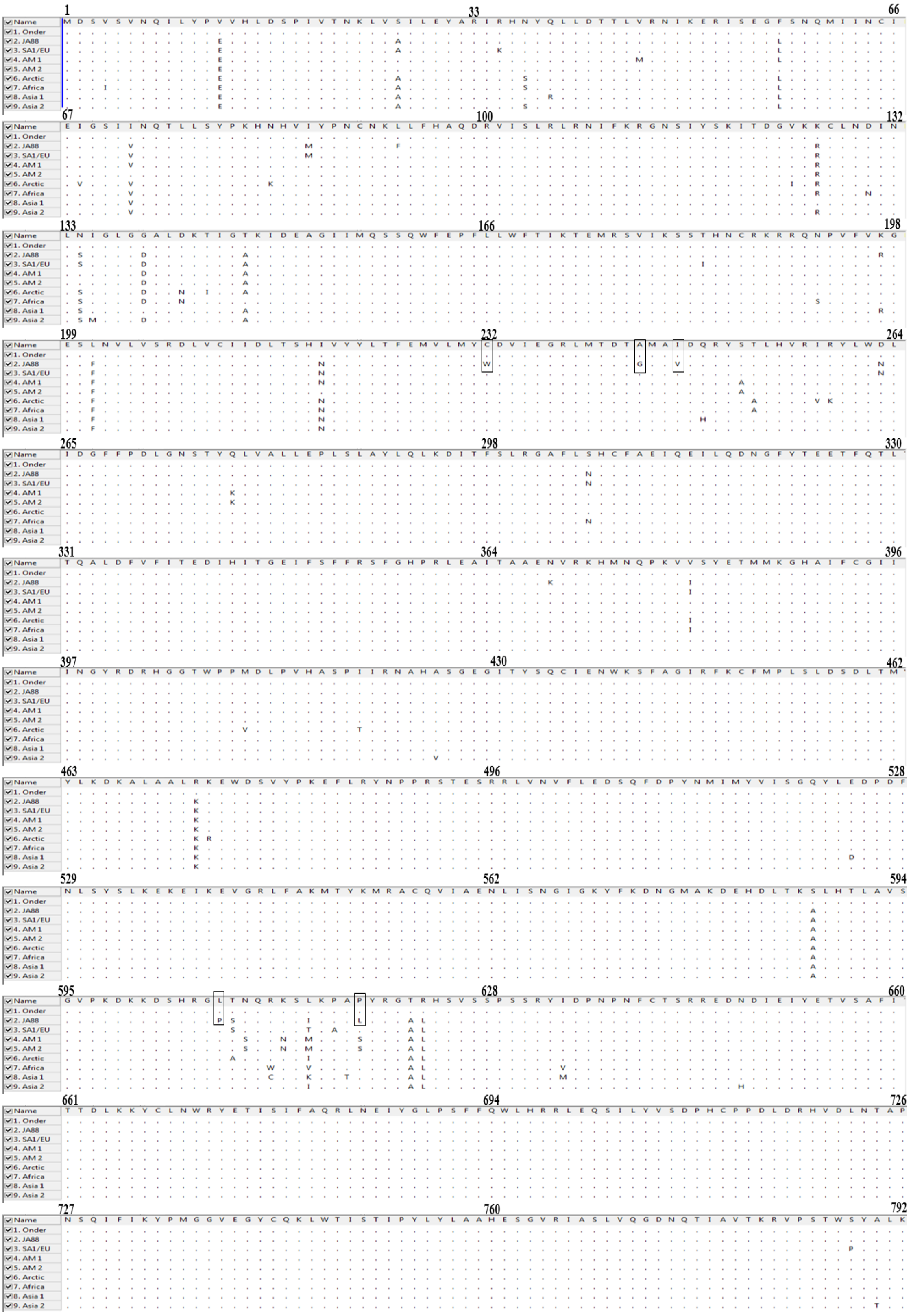

Fig. S4. Multiple amino acid sequence alignment of the Large protein (Part I). The sequences were compared by Clustal Multiple Sequence Alignment Program software. The boxes indicate amino acid residues unique to JA88 strain. Dots (•) indicate identity.

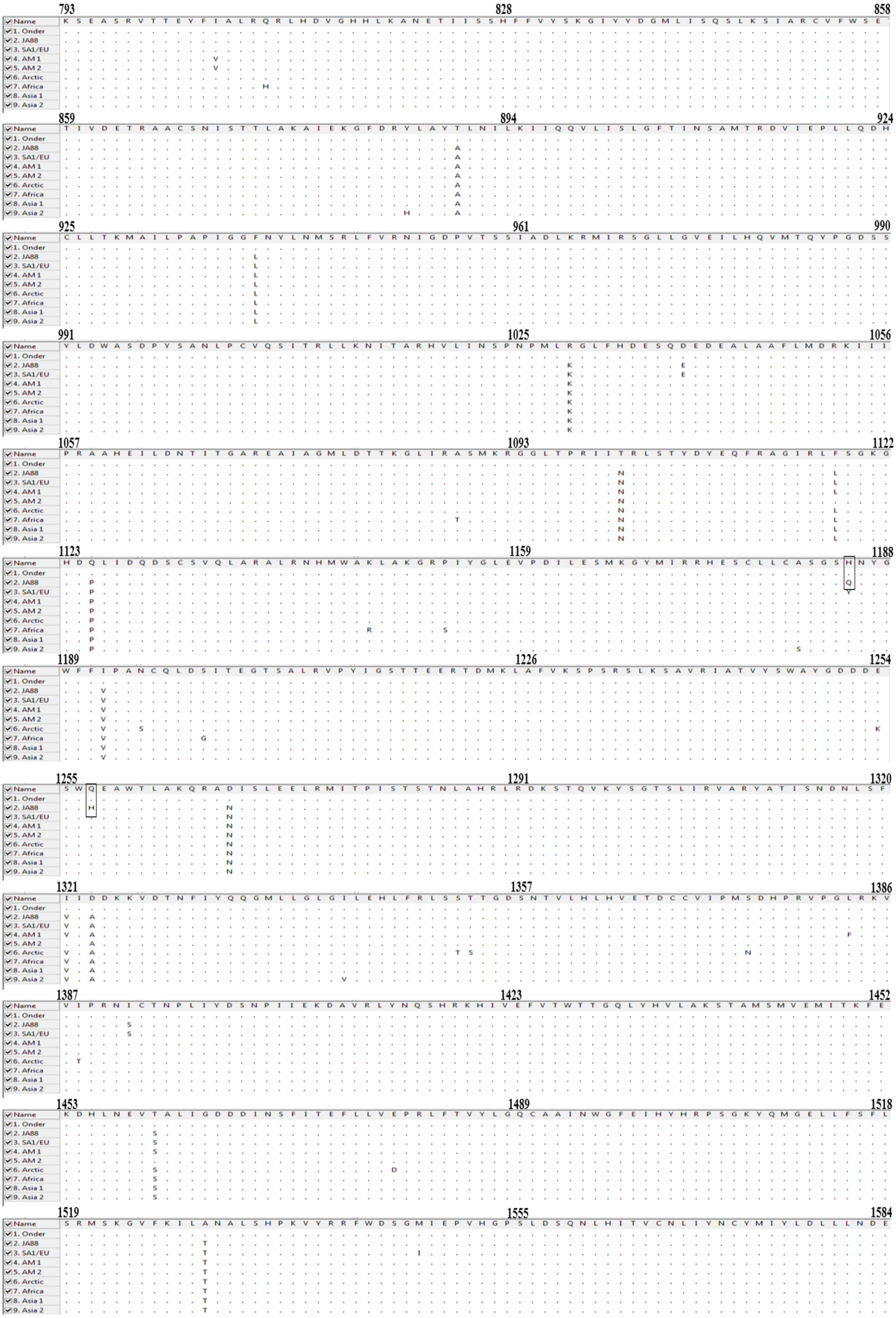

Fig. S4. Multiple amino acid sequence alignment of the Large protein (Part II). The sequences were compared by Clustal Multiple Sequence Alignment Program software. The boxes indicate amino acid residues unique to JA88 strain. Dots (•) indicate identity.

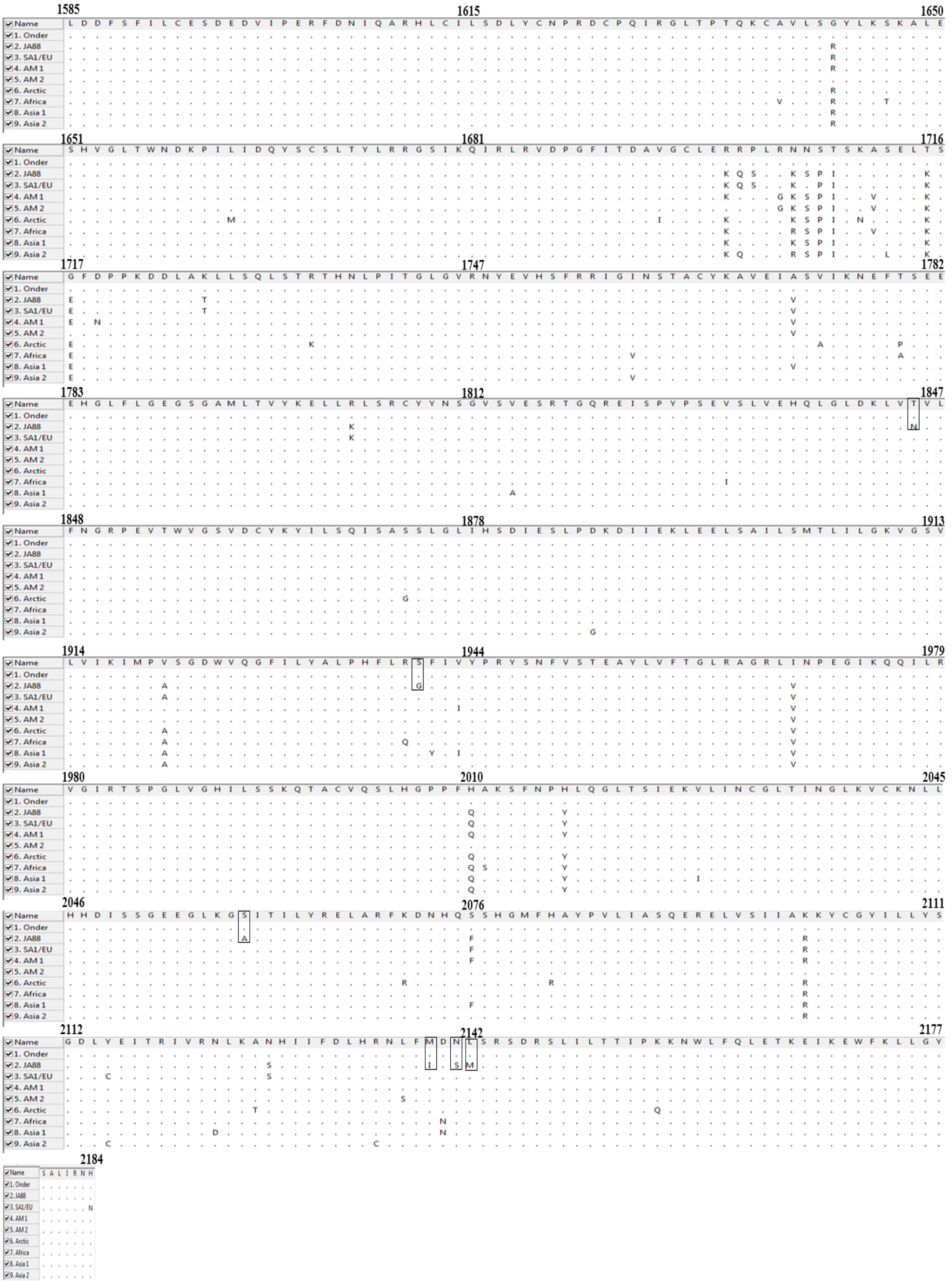

Fig. S4. Multiple amino acid sequence alignment of the Large protein (Part III). The sequences were compared by Clustal Multiple Sequence Alignment Program software. The boxes indicate amino acid residues unique to JA88 strain. Dots (•) indicate identity.
